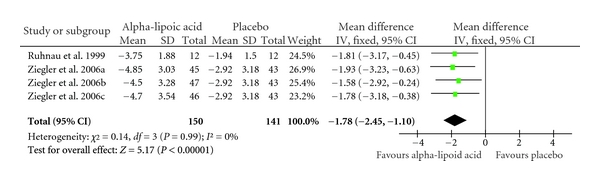# Alpha Lipoic Acid for Symptomatic Peripheral Neuropathy in Patients with Diabetes: A Meta-Analysis of Randomized Controlled Trials

**DOI:** 10.1155/2012/456279

**Published:** 2012-01-26

**Authors:** Gerritje S. Mijnhout, Boudewijn J. Kollen, Alaa Alkhalaf, Nanno Kleefstra, Henk J. G. Bilo

**Affiliations:** ^1^Department of Internal Medicine, Isala Clinics, P.O. Box 10400, 8000 GK Zwolle, The Netherlands; ^2^Department of General Practice, University Medical Centre Groningen, University of Groningen, P.O. Box 30001, 9700 RB Groningen, The Netherlands; ^3^Department of Internal Medicine, University Medical Centre Groningen, University of Groningen, P.O. Box 30001, 9700 RB Groningen, The Netherlands; ^4^Diabetes Centre, Isala Clinics, P.O. Box 10400, 8000 GK Zwolle, The Netherlands; ^5^Langerhans Medical Research Group, P.O. Box 21, 4254 ZG Sleeuwijk, The Netherlands

## Abstract

*Objective*. We performed a systematic review of the literature to evaluate the effects of alpha lipoic acid for symptomatic peripheral neuropathy in patients with diabetes mellitus. *Research design and methods*. The databases MEDLINE and EMBASE were searched using the key words “lipoic acid”, “thioctic acid”, “diabet^∗^”, and the MeSH-terms “thioctic acid” and “diabetes mellitus”. Randomised controlled trials using the TSS score as the outcome measure were selected and assessed for their methodological quality. Study selection and quality assessment were performed independently by three observers. *Results*. Overall, the pooled standardized mean difference estimated from all trials revealed a reduction in TSS scores of −2.26 (CI: −3.12 to −1.41; *P* = 0.00001) in favour of alpha lipoic acid administration. Subgroup analyses of oral administration (−1.78 CI: −2.45 to −1.10; *P* = 0.00001) and intravenous administration (−2.81 CI: −4.16 to −1.46; *P* = 0.0001) confirmed the robustness of the overall result. *Conclusions*. When given intravenously at a dosage of 600 mg/day over a period of 3 weeks, alpha lipoic acid leads to a significant and clinically relevant reduction in neuropathic pain (grade of recommendation A). It is unclear if the significant improvements seen after 3–5 weeks of oral administration at a dosage of >600 mg/day are clinically relevant.

## 1. Introduction

Neuropathy is a microvascular complication of diabetes mellitus which leads to considerable morbidity and a decreased quality of life [1]. Peripheral neuropathy can present as tingling, burning, pain, cramps, paresthesia, or numbness. There is overwhelming evidence that the development of microvascular complications is related to the level of glucose dysregulation over a long period of time [2]. Hyperglycaemia induces an increased production of free oxygen radicals in the mitochondria (oxidative stress), which leads to the activation of the four known pathways that are responsible for hyperglycaemic damage: the polyol, hexosamine, protein kinase C, and AGE pathways [3]. This results in damage of endothelial and neuronal cells. 

Neuropathic pain is difficult to treat, and standard analgesics are usually not effective enough [4]. The medications which are currently used to treat neuropathic pain in patients with diabetes include mainly antidepressants, antiepileptics, and opioids. These medications are limited in their effectiveness, they have considerable side effects, and they have no effect on the processes by which hyperglycaemia leads to cell damage [5]. Antioxidants, such as alpha lipoic acid, could theoretically be effective in treating diabetic neuropathy. In 1951, alpha lipoic acid was identified as a coenzyme in the tricarboxylic acid cycle (Krebs Cycle) [6]. Alpha lipoic acid is also a potent antioxidant, reported to reduce and prevent diabetic micro- and macrovascular complications in animal models [7, 8]. A recent study in humans with type 1 diabetes mellitus showed a normalisation of the increased AGE formation and a reduction of the hexosamine pathway [9]. By preventing the damage caused by hyperglycaemia, alpha lipoic acid may not only be an analgesic treatment but may also improve nerve function. In addition, recent evidence shows that alpha lipoic acid decreases neuronal sensitivity to pain by selectively inhibiting neuronal T-type calcium channels [10]. Moreover, compared to the medications currently in use, alpha lipoic acid has few side effects [11]. In Germany, alpha lipoic acid is approved for the treatment of diabetic neuropathic pain and covered by health insurance companies, but use has not been widely adopted elsewhere.

An earlier meta-analysis of four randomized controlled trials (RCTs) on alpha lipoic acid (600 mg/day) in patients with diabetes and neuropathic pain concluded that three weeks of treatment with intravenous alpha lipoic acid (600 mg/day) led to a significant decrease in reported neuropathic pain [12]. However, studies investigating the effect of oral administration were not included. In addition, the meta-analysis did not fulfil the Cochrane methodological criteria for systematic reviews. A protocol for a proposed systematic review can be found in the Cochrane Library [13]. Recently, we performed a qualitative systematic review of the literature [14]. In addition, it was our purpose to extend the literature search and to perform a quantitative meta-analysis. The aim of this meta-analysis was to evaluate the effects of intravenous as well as oral administration of alpha lipoic acid versus placebo in patients with symptomatic peripheral diabetic neuropathy.

## 2. Research Design and Methods

### 2.1. Literature Search

In November 2010, three of the authors (GSM, AA, and NK) conducted a search for relevant publications in the electronic database MEDLINE, using the search engine *PubMed*, and EMBASE. The search strategy used in MEDLINE used the terms “lipoic acid”, “thioctic acid”, and “diabet*” and the MeSH terms “thioctic acid” and “diabetes mellitus”: (((lipoic acid OR thioctic acid OR thioctic acid [MeSH]) AND (diabete* OR diabeti* OR diabeto* OR diabetes mellitus [MeSH])) AND ((clinical [Title/Abstract] AND trial [Title/Abstract]) OR clinical trials [MeSH Terms] OR clinical trial [Publication Type] OR random*[Title/Abstract] OR random allocation [MeSH Terms] OR therapeutic use [MeSH Subheading])). A similar search strategy was used in EMBASE: ((lipoic acid OR thioctic acid) AND (diabetes mellitus OR diabetic*) AND ([cochrane review]/lim OR [controlled clinical trial]/lim OR [meta analysis]/lim OR [randomized controlled trial]/lim OR [systematic review]/lim)). All authors obtained the same results.

### 2.2. Study Selection

For study selection, the following inclusion criteria were used: (1) RCTs on alpha lipoic acid, (2) a study population consisting of patients with diabetes mellitus and peripheral neuropathic pain, and (3) use of the total symptom score (TSS) as the outcome measure. Language was not a restriction. GSM, AA, and NK independently identified studies to be included in the review by checking the titles and abstracts downloaded from the databases. A consensus meeting was then held to resolve any disagreements. The final decision to include or exclude any study was based on the article's full text. The reference lists of the identified studies were reviewed to discover additional potentially eligible studies. Unpublished data and conference proceedings were excluded from this review.

### 2.3. Methodologic Quality Assessment

The aforementioned authors proceeded to independently evaluate the quality of each study using the standardised evaluation form for RCTs and systematic reviews developed by the Dutch Cochrane Centre (http://www.cochrane.nl/) (Table 1). Levels of evidence and recommendation grades were applied according to the Oxford Centre of Evidence-based Medicine, version 2001 (http://www.cebm.net/index.aspx?o=1025/).

### 2.4. Outcome Measure

The primary outcome measure in this meta-analysis was the total symptom score (TSS). The TSS is a questionnaire in which the patient is asked to assess the intensity (absent, mild, moderate, severe) and the frequency (now and then, often, continuous) of four symptoms (pain, burning, paresthesia, numbness) resulting in a scaled score in which 0 means no symptoms and 14.64 means that all four symptoms are severe and more or less continuously present (Table 2). A 30% change on this scale is considered to be clinically relevant (or ≥2 points in patients with a starting score ≤4 points) [15].

### 2.5. Statistical Analysis

For the purpose of this meta-analysis, overall results based on TSS scores were combined for oral and intravenous administration of alpha lipoic acid and placebo. Meta-analysis was undertaken using RevMan5 software (The Nordic Cochrane Centre, The Cochrane Collaboration). The *I* ^2^ statistic was used to assess statistical heterogeneity [19]. An *I* ^2^ > 30% was considered to denote heterogeneity. A random-effect model was used in case of heterogeneity, a fixed-effect model in the absence of heterogeneity. The inverse-variance method was used to weigh the scores of individual studies. When possible, study authors were contacted to clarify data. Studies were excluded from the meta-analysis if insufficient information was provided to enable standard error calculation. The Mantel–Haenszel method was subsequently applied to estimate pooled effect sizes. In order to explore the robustness of our results we conducted the following, a priori specified, subgroup analyses: intravenous and oral administration of alpha lipoic acid versus placebo.

We adhered to the QUOROM guidelines for the reporting of meta-analyses of randomised trials [20].

## 3. Results

### 3.1. Identification and Selection of Studies

**Figure 1:**
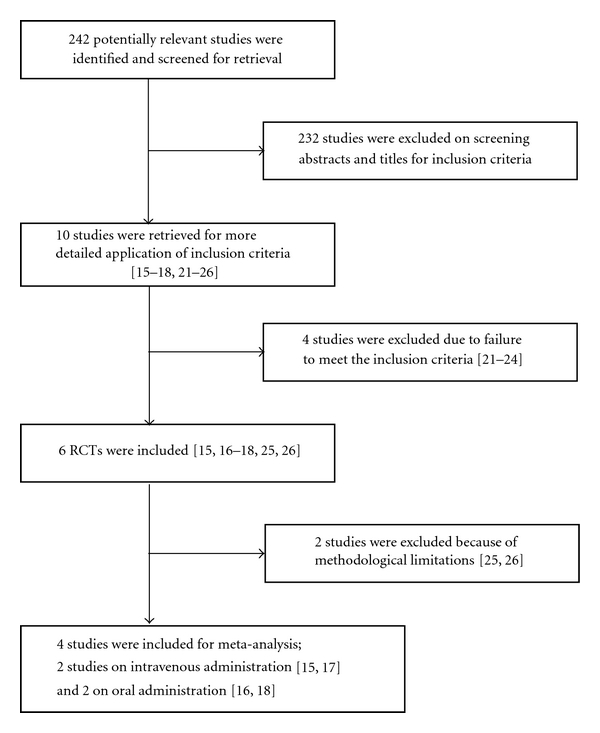
Flow diagram.

The search yielded 242 publications in Medline and 112 in Embase (Figure 1). The 112 publications found in Embase were also identified in Medline. After reviewing the titles and the abstracts of the 242 publications, 10 randomised placebo-controlled trials on alpha lipoic acid in patients with diabetic neuropathic pain were selected [15–18, 21–26]. After reading the complete articles, two studies were excluded [21, 22], because they dealt with the effects of alpha lipoic acid on autonomic instead of diabetic neuropathy. Two studies [23, 24] were excluded because the TSS was not used as an outcome measure. There was no disagreement among the reviewers regarding the studies selected for inclusion.

### 3.2. Methodological Quality Assessment

A survey of the methodological quality assessment is shown in Table 1. Four of the RCTs [15–18] were of good methodological quality (level 1b). Two RCTs [25, 26] had substantial methodological limitations (level 2b). The study of Liu et al. [26] was excluded from our meta-analysis because of unacceptable methodological limitations, including absence of allocation concealment and blinding. The study of Ziegler et al. [25] was considered for inclusion despite exclusion bias due to selective loss to follow-up, but the article provided insufficient information to enable standard error calculation. The study authors were contacted to clarify data, but they did not respond to repeated requests. Therefore, also this study was excluded from the meta-analysis. 

### 3.3. Descriptive Analyses of Selected Randomized Controlled Trials

Finally, four RCTs were included in our systematic review and meta-analysis. The study populations in the four selected RCTs were all made up of patients with peripheral diabetic neuropathy [15–18]. The age range was from 18 to 74 years, and most of the patients included had type 2 diabetes mellitus. The effects of orally administered alpha lipoic acid were investigated in two studies and intravenous administration in another two studies (Table 3). Two studies incorporated multiple dose comparisons. The dosage of alpha lipoic acid ranged from 100 to 1800 mg per day. Intravenous alpha lipoic acid was given for three weeks, and oral administration varied between three weeks and six months. 

A significant improvement in the TSS scores was reported in all studies. In these studies an average 50% reduction was seen in the TSS with the oral or intravenous administration of at least 600 mg per day. However, when compared to the subjects in the control groups, the reduction in TSS was actually less than the clinically relevant threshold of 30% [15], as the TSS in the control group also decreased. This was particularly evident in the studies where alpha lipoic acid was administered orally. In one study, in which the alpha lipoic acid was administered intravenously, the intervention group did show a more than 30% reduction in TSS when compared to the control group [16]. Dosages higher than 600 mg per day did not result in a further improvement in the TSS and resulted in a greater incidence of side effects such as nausea, vomiting, and dizziness. The side effects seen with dosages ≤600 mg per day were not different than seen with placebo. A safety analysis of treatment with alpha lipoic acid over 4 years in diabetic polyneuropathy [27] showed that treatment tolerability and discontinuations due to lack of tolerability did not differ between placebo and treatment groups. However, the rates of serious adverse events were higher on alpha lipoic acid (38.1%) than those on placebo (28.0%) [27]. Of all reported adverse events, only heart rate and rhythm disorders were observed significantly more frequently in patients treated with alpha lipoic acid compared to patient treated with placebo (6.9% versus 2.7%, *P* 0.047) [27]. 

### 3.4. Meta-Analysis

Overall, the pooled standardized mean difference estimated from all trials revealed a reduction in TSS scores of −2.26 (CI: −3.12 to −1.41; *P* = 0.00001) in favour of alpha lipoic acid administration (Table 4). The outcome of the subgroup analyses of oral administration (−1.78 CI: −2.45 to −1.10; *P* = 0.00001) and intravenous administration (−2.81 CI: −4.16 to −1.46; *P* = 0.0001) confirmed the robustness of the overall result (Tables 5 and 6).

## 4. Discussion

Based on the four level 1b randomized, placebo-controlled studies included here, there is evidence to support that alpha lipoic acid causes a significant and clinically relevant decrease in neuropathic pain when administered for a period of three weeks at a dosage of 600 mg per day (grade of recommendation A). However, the significant improvements seen after the oral administration of alpha lipoic acid over a period of 3–5 weeks at a dosage of ≥600 mg per day are probably not clinically relevant, because the reduction in TSS was actually less than the threshold of 30% considered to be clinically relevant. There are, at present, no publications in which the effects of long-term treatment with intravenous or oral lipoic acid are presented. 

The RCTs are primarily performed by a single German research group. A number of these studies were multicenter studies which included German as well as Russian, Israeli, and Croatian patients. Presumably, there is no overlap between these patient populations. All studies were sponsored by a pharmaceutical company which manufactured alpha lipoic acid. A number of the authors received salaries from this company, besides which, the pharmaceutical company also had representatives sitting on the advisory body for several of these studies. 

It is striking that clinically relevant effects on neuropathic pain are seen after only 3–5 weeks of alpha lipoic acid administration. This is unexpectedly rapid for an antioxidising diet supplement. This may be explained by the selective modulation of neuronal T-type calcium channels by alpha lipoic acid [10]. In studies on diabetic autonomic neuropathy, effects of alpha lipoic acid were seen after 8–16 weeks [21, 22], depending on the study design.

The included RCTs were not designed for neuropathic pain. Individual scores on each of the four symptoms of the TSS (pain, burning, paresthesia, numbness) were not available from the included studies. 

Unfortunately, there are not yet any results published for its administration over a longer time period. The continued, long-term effectiveness of any treatment is of the utmost importance for chronic conditions such as diabetic neuropathy. 

In The Netherlands, the cost of using alpha lipoic acid at a dosage of 600 mg per day varies between 17.15 and 75.00 euros per month, depending on the manufacturer [14]. In comparison, the costs of amitriptyline, carbamazepine, duloxetine, gabapentin, and pregabalin are, respectively, 3.41, 9.38, 35.80, 53.75, and 71.71 euros per month (based on the Z-index tax, 2010) [28].

Finally, a meta-analysis is likely to suffer from publication bias, methodological deficiencies, and heterogeneity. We kept the likelihood of bias to a minimum by developing a detailed protocol before starting this study, undertaking a meticulous search for published studies, and using explicit methods for study selection, data extraction, and data analysis. Also, we studied the totality of the randomized evidence by including all relevant properly randomized trials.

We conclude that intravenous administration of alpha lipoic acid leads to significant and clinically relevant improvements of symptomatic peripheral diabetic neuropathy in the short term. The results we present are encouraging enough to consider intravenous alpha lipoic acid for the treatment of diabetic neuropathy in patients, who do not respond to common therapy. It is unclear if the significant improvements seen with the oral administration of alpha lipoic acid are clinically relevant. Additional research of longer duration using an informative neuropathic pain scale will be necessary to investigate the effects of both routes.

## Figures and Tables

**Table 1:** Methodological quality assessment of the included intervention studies.

		Ziegler 1995 [15] ALADIN	Ruhnau 1999 [16] ORPIL	Ametov 2003 [17] SYDNEY	Ziegler 2006 [18] SYDNEY 2
(1)	Randomisation?	yes	yes	yes	yes
(2)	Concealment of allocation?	yes	yes	yes	yes
(3)	Patients blinded?	yes	yes	yes	yes
(4)	Doctors blinded?	yes	yes	yes	yes
(5)	Investigators blinded?	NO	NO	NO	NO
(6)	Groups comparable at baseline?	yes	yes	yes	yes
(7)	Follow-up complete of >80% of patients?	yes	yes	yes	yes
(8)	Intention-to-treat analysis?	yes	yes	yes	yes
	Level of evidence	1b	1b	1b	1b

**Table 2:** Total Symptom Score (TSS): scoring system for neuropathic symptoms (pain, burning, paresthesia, and numbness). The score can range from 0 (no symptoms) to maximally 14.64 (all symptoms present, severe, continuous).

Symptom frequency	Symptom intensity			
	Absent	Slight	Moderate	Severe
Occasional	0	1.00	2.00	3.00
Frequent	0	1.33	2.33	3.33
(Almost) continuous	0	1.66	2.66	3.66

**Table 3:** Overview of the included randomized, placebo-controlled studies with alpha lipoic acid in persons with symptomatic peripheral diabetic neuropathy.

Study 1st author, year; study name	Research group		Length of study	Alpha lipoic acid dosage	Administration route	Primary outcome measure	Findings		Difference intervention versus control* (Significance)	level of evidence
	Patient type	Number of patients (Intervention/control)					Intervention	Control		
Ziegler 1995 ALADIN [15]	DM2; 18–70 yr	328 (65/63/66/66)	3 weeks	(a) 100 mg daily (b) 600 mg daily (c) 1200 mg daily	Intravenous	TSS	(a) 7.6 → 4.3 (b) 7.8 → 2.8 (c) 7.6 → 3.1	6.8 → 4.2	−0.7 (ns) −2.4 (*P* < 0.001) −1.9 (*P* = 0.003)	1b
Ruhnau 1999 ORPIL [16]	DM2; 18–70 yr	24 (12/12)	3 weeks	3dd600 mg	Oral	TSS	7.99 → 4.24	8.18 → 6.24	−1.81 (*P* = 0.021)	1b
Ametov 2003 SYDNEY [17]	DM1+ DM2; 18–74 yr	120 (60/60)	3 weeks	600 mg daily for 14 days	Intravenous	TSS	−5.72	−1.83	−3.89 (*P* < 0.001)	1b
Ziegler 2006 SYDNEY 2 [18]	DM1+ DM2; 18–74 yr	181 (45/47/46 /43)	5 weeks	(a) 600 mg daily (b) 1200 mg daily (c) 1800 mg daily	Oral	TSS	(a) 9.44 → 4.59 (b) 9.40 → 4.90 (c) 9.02 → 4.32	9.27 → 6.35	−1.93 (*P* < 0.05) −1.58 (*P* < 0.05) −1.78 (*P* < 0.05)	1b

*Calculated differences between intervention and control groups: not controlled.

DM: diabetes mellitus.

ns: not significant.

TSS: Total Symptom Score.

**Table 4:** Standardized mean differences for the administration of orally and intravenously administered alpha-lipoic acid versus placebo in the treatment of neuropathic pain. Diamond denotes pooled estimate of overall effect. Weighing of individual studies is based on the inverse variance method. For subgroups, see Table 3.

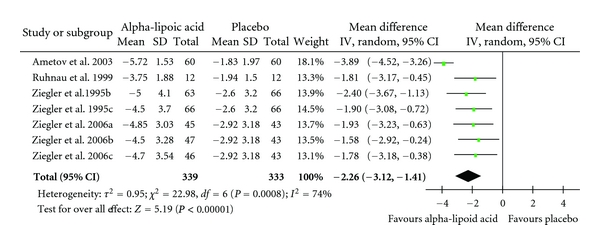

**Table 5:** Standardized mean differences for the administration of intravenously administered alpha-lipoic acid versus placebo in the treatment of neuropathic pain. Diamond denotes pooled estimate of overall effect. Weighing of individual studies is based on the inverse variance method. For subgroups, see Table 3.

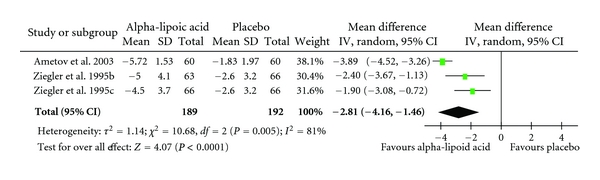

**Table 6:** Standardized mean differences for the administration of orally administered alpha-lipoic acid versus placebo in the treatment of neuropathic pain. Diamond denotes pooled estimate of overall effect. Weighing of individual studies is based on the inverse variance method. For subgroups, see Table 3.